# Three New Species of *Deltoblastus* Fay from the Permian of Timor

**DOI:** 10.1371/journal.pone.0127727

**Published:** 2015-06-10

**Authors:** Ryan FitzGerald Morgan

**Affiliations:** 1 Department of Chemistry, Geosciences, and Physics, Tarleton State University, Stephenville, Texas, United States of America; 2 Department of Geology, Baylor University, Waco, Texas, United States of America; Duke University Marine Laboratory, UNITED STATES

## Abstract

*Deltoblastus* is a genus of Permian blastoid comprised of 15 species, each differing based on subtle thecal morphology differences. Three new species are introduced here, based on characteristics present which distinguish individuals from established morphotypes. In order to guarantee a more complete understanding of the genus, a complex character matrix containing all 15 named and three new species was created, defining all species based on the presence or absence of 30 unique traits. Differences in character compositions give evidence for unique thecal morphologies, supporting the three new species which are proposed.

## Introduction

Blastoids are a class of extinct marine echinoderms, characterized by five-fold symmetry, small columnar stems, and small theca. *Deltoblastus* Fay is a genus of Schizoblastid blastoid, composed of 15 species, characterized by elongate deltoid plates and diminished basal plates which are often invaginated. *Deltoblastus* is one of many radiating blastoid genera in the Permian, and the only member of family Schizoblastidae outside of the Early Carboniferous [1]. Recent investigation into the collections at Baylor University and the Natural History Museum of London (NHMUK) yields *Deltoblastus* specimens not ascribable to named species due to significant differences in theca morphology. These specimens are herein assigned to new species within *Deltoblastus*, and description of these is the purpose of this paper.

## Geologic Setting

*Deltoblastus* is a Permian genus, primarily constrained to the island of Timor, with a few examples from Australia, Oman, and eastern Russia. The Permian *Deltoblastus* assemblages range from the Early to Middle Permian [2, 3, 4] and the abundance of this genus, particularly in the Timorese deposits, likely represents unique conditions favorable to *Deltoblastus* proliferation, as numbers of preserved Deltoblastus far exceed expected if only preservation effects were at work [2]. The *Deltoblastus* species described in this paper originate from Permian Timorese deposits. These deposits are heterogeneous, and have presented a complex problem for geologists for decades. Recent interpretations suggest this region is dominated by a shallow mixed marine carbonate and volcanics succession, while in some areas it is a thin bedded siliciclastic succession [2]. While traditional interpretations have placed the carbonate and volcanic succession as allochthonous, recent revision places both successions as autochthonous within a lithologically heterogeneous basin complex [2].

## Methods

**Fig 1:**
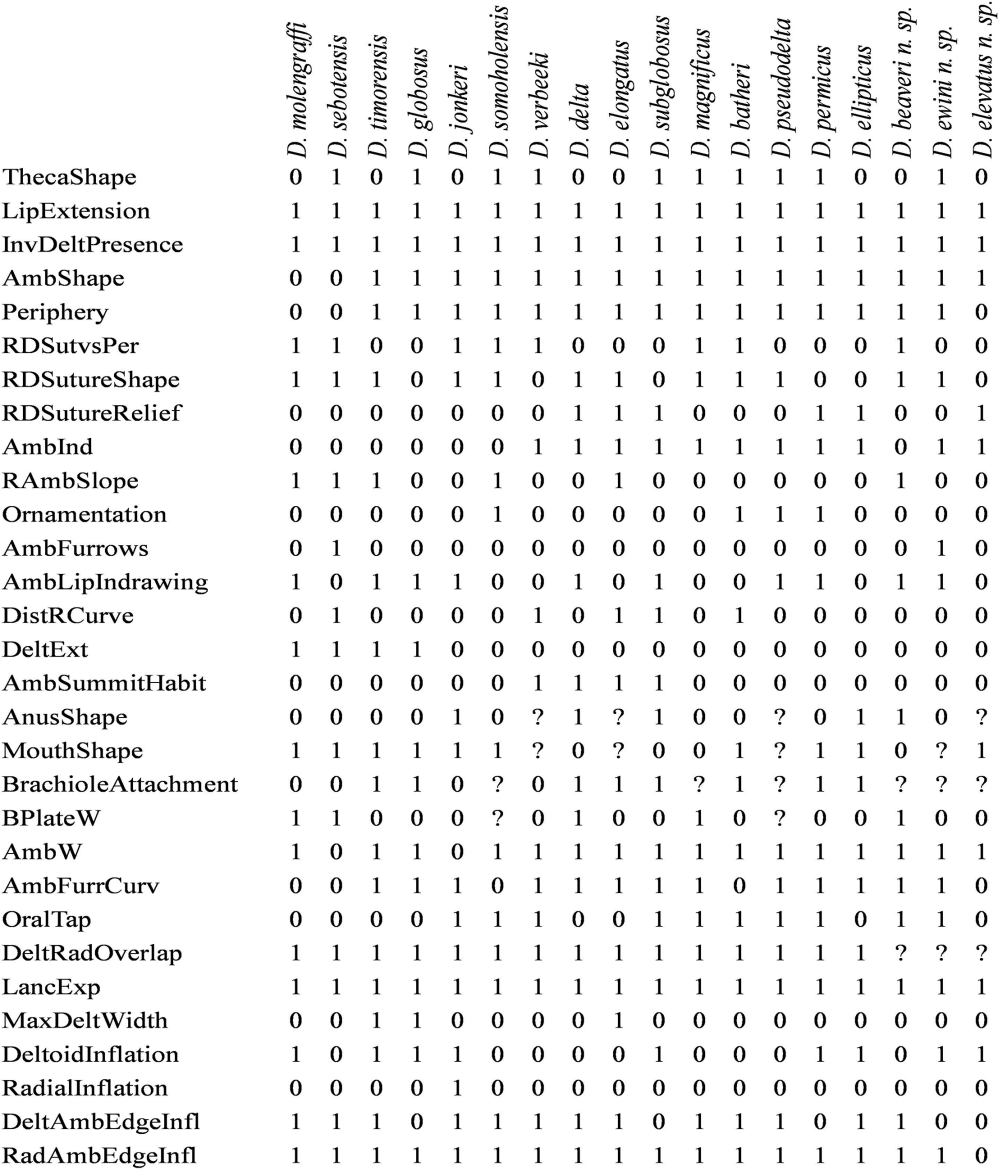
Table of *Deltoblastus* binomial character matrix. See remarks for a thorough comparison of thecal characters of proposed new species to established species. Black = 1, white = 0. RDSutureRelief = Relief on the thecal surface of the radiodeltoid suture, 1 = depressed, 0 = flush/no relief; AmbSummitHabit = shape of the ambulacral groove at summit truncation, 1 = pointed/rounded, 0 = linear; AnusShape = shape of the anal opening, 1 = triangular, 0 = ovoid; AmbInd = indentation of the ambulacral groove, 1 = depressed, 0 = flush; MaxDeltWidth = position of the maxium width of the deltoid plate, 1 = above radiodeltoid suture, 0 = at the radiodeltoid suture; BrachioleAttachment = shape of the brachiole attachment point, 1 = pointed, 0 = ovoid; AmbFurrCurv = curvature of the ambulacral furrow, 1 = curved, 0 = straight; AmbW = ambulacral width, 1 = medium/wide, 0 = thin; AmbShape = ambulacral groove shape, 1 = lancet, 0 = linear; AmbLipIndrawing = severity of indrawing of the ambulacra at the radial lip, 1 = significant, 0 = slight/none; Periphery = position of the periphery on the theca, 1 = low/adoral, 0 = high/oral; DeltRadOverlap = presence of overlap of the deltoids/radials, 1 = present, 0 = absent; DeltoidInflation = inflation of the deltoid plate, 1 = significant inflation, 0 = none; LipExtension = extension of the radial lip adorally, 1 = extended, 0 = none; InvBasPresence = invagination of the basal plates, 1 = invaginated, 0 = none/protruding; RadAmbEdgeInfl = inflation of the radial plate along the contact with the ambulacral groove, 1 = inflated, 0 = depressed/inverted; LancExp = exposure of the lancet, 1 = exposed, 0 = not exposed; ThecaShape = overall shape of the theca in profile view, 1 = globose, 0 = ovoid; DeltAmbEdgeInfl = inflation of the deltoid plate along the contact with the ambulacral groove, 1 = inflated, 0 = none; OralTap = tapering of plates approaching the oral surface, 1 = severe, 0 = slight/gradual; DistRCurve = curvature of the distal (adoral) end of the radial plate, where flat and concave growth of this plate is the most common observed outside this genus, 1 = flat/convex, 0 = concave; MouthShape = shape of the oral openings, 1 = slit, 0 = ovoid; RDSutureShape = shape of the radiodeltoid suture, 1 = pointed, 0 = horizontal; Ornamentation = presence of theca ornamentation, 1 = ornamented, 0 = smooth; BPlateW = width of the basal plates, 1 = wide, 0 = small; RDSutvsPer = position of the radiodeltoid suture versus position of the periphery, 1 = suture at or above periphery, 0 = suture below periphery; RAmbSlope = degree of incline between radial plate and ambulacral groove, 1 = shallow/horizontal, 0 = steep; DeltExt = extension of the deltoids above the oral surface, 1 = no extension, 0 = ridge/comb/point; RadialInflation = inflation of the radial plate, 1 = inflated, 0 = depressed/flat; AmbFurrows = number of ambulacral furrows in 5mm, 1 = >14, 0 = ≤14.

Investigation into these *Deltoblastus* species involved a detailed study of important thecal characters presented by the specimens. Initial visualization of the defining characters separating *Deltoblastus* species was attained using a detailed presence-absence character matrix, whose construction was based on information from original species descriptions and plates [5, 6, 7, 8, 9] and the suspected new species were added to this matrix (Fig 1). Further evaluation of species of merit was attained from individual examination and comparison to electrotypes, published descriptions, and detailed figures. Character choices were based on observed attributes and those defined as unique to species by previous researchers [5, 6, 7, 8, 9]. No permits were required for the described study, which complied with all relevant regulations. Specimen coating (where applied) was performed via ammonium chloride sublimation.

### Nomenclatural Acts

The electronic edition of this article conforms to the requirements of the amended International Code of Zoological Nomenclature, and hence the new names contained herein are available under that Code from the electronic edition of this article. This published work and the nomenclatural acts it contains have been registered in ZooBank, the online registration system for the ICZN. The ZooBank LSIDs (Life Science Identifiers) can be resolved and the associated information viewed through any standard web browser by appending the LSID to the prefix "http://zoobank.org/". The LSID for this publication is: urn:lsid:zoobank.org:pub:E4D8639C-95B8-4F21-8B1F-E45C2342AFF9. The electronic edition of this work was published in a journal with an ISSN, and has been archived and is available from the following digital repositories: PubMed Central, LOCKSS. Specimens studied can be found at the following repositories: National Museum of Natural History, Smithsonian Institution: USNM 594945, USNM 594946, USNM 594947; Natural History Museum of London: NHMUK e59727, e59734, e59209, e59210, and e59212.

## Systematic Paleontology

Class Blastoidea Say, 1825 [10]

Family Schizoblastidae Etheridge and Carpenter, 1886 [11]

Genus *Deltoblastus* Fay, 1961 [5]

*Type species*.*—Schizoblastus delta* var. *elongata* Wanner, 1924 [7]

***Deltoblastus beaveri*** new species

urn:lsid:zoobank.org:act:CD3D10DA-D55E-42E5-9279-D21344BDA107

(Fig 2A–2C)

**Fig 2:**
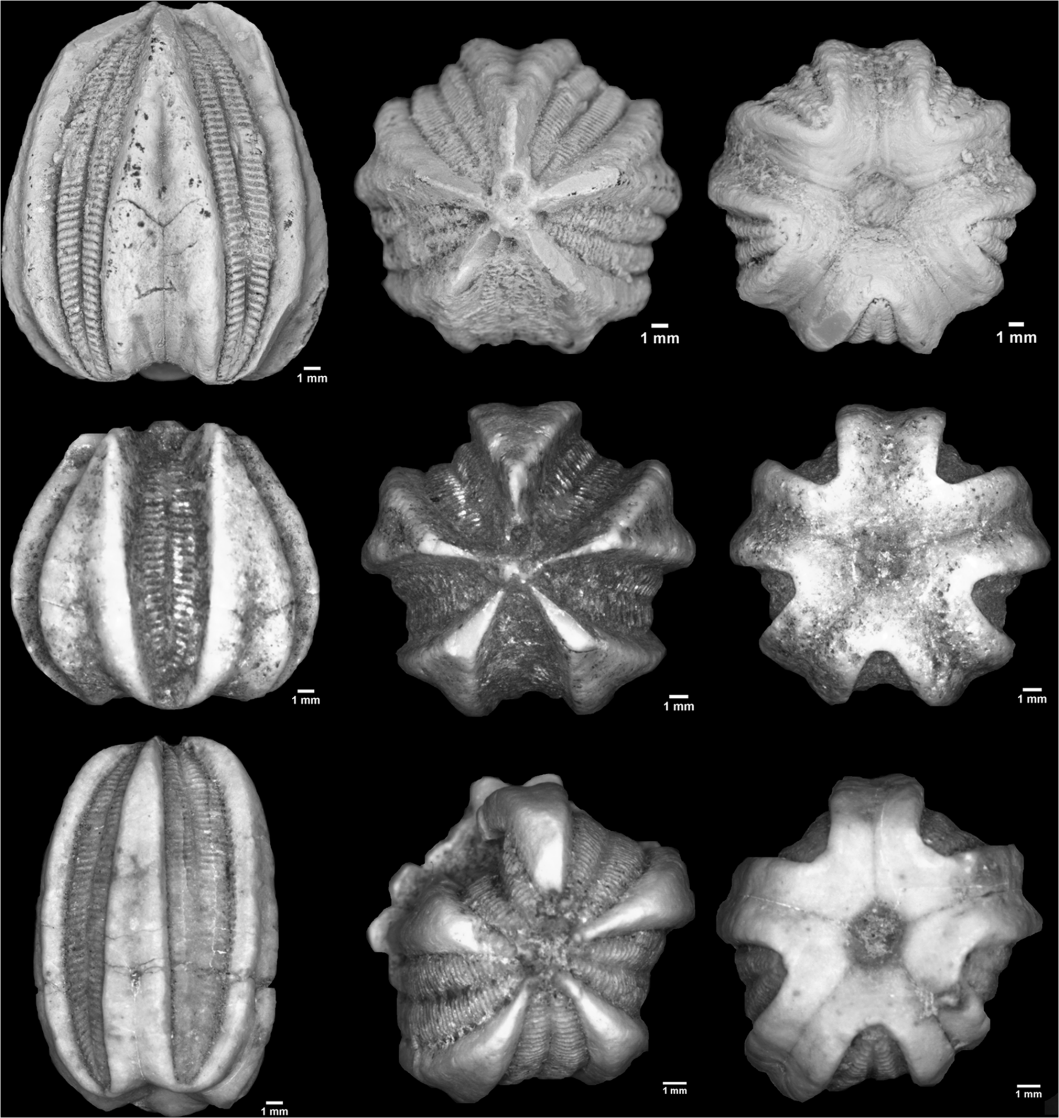
Staged photographs of new *Deltoblastus* species. Top) *Deltoblastus beaveri* sp. nov (left-to-right: theca, oral, and basal views), coated via ammonium chloride sublimation to maximize visibility of features; Middle) *Deltoblastus ewini* sp. nov (left-to-right: theca, oral, and basal views), uncoated, as per NMUK requirements; Bottom) *Deltoblastus elevatus* sp. nov (left-to-right: theca, oral, and basal views), uncoated, as per NMUK requirements.

### Derivation of name

Named for Dr. Harold H. Beaver, professor emeritus, Baylor University.

### Type specimen

USNM 594945.

### Diagnosis

*Deltoblastus beaveri* sp. nov. possesses a subellipsoidal calyx, higher-than-wide. Large, rounded triangular anal opening, separate from small ovoid oral openings. Prominent deltoid septa are broken in holotype, appearing to extend into a ridge or comb structure. The deeply invaginated basal plates are wide for *Deltoblastus*, and distal radial curve approaching basals is convex in shape. Elongate ambulacra are wide and lancet shaped, in a shallow depression. Ambulacral lips are strongly indrawn, emphasizing the convex radial curve approaching the basal plates. Radials are extended, exceeding the length of deltoids, an oddity for *Deltoblastus*. Radiodeltoid suture is flush to theca and is v-shaped, pointing distally. No ornamentation is observable, barring growth striae. Subdued deltoid ridges with minor inflation along length of ambulacra, with a shallow slope to the ambulacral groove. Ambulacral furrows number 10–12 in 5mm length, and are slightly arcuate in shape.

### Description

Subellipsoidal theca, lacking ornamentation. Growth striae present, but shallow and hard to discern. Deeply invaginated basal plates. Cross-section is roughly a rounded decagon, with indented ambulacral groves. Theca height ~1.2 times that of theca width at greatest points. Radial length exceeds that of deltoid length. Deltoid 1.5 times wider than an ambulacrum at radiodeltoid suture. Ambulacra indented and wide, with ten to twelve furrows in 5mm length. Ambulacra length greater than two times the deltoid length, and nearly the length of the theca. Radials and deltoids inflated along ambulacrum edges, adorally becoming shallow crests or peaks. Radio-deltoid suture pointed basally, with theca periphery well below suture.

The small basal plates form a steep sided, truncated cone (Fig 2C). Invagination depth is 1/5^th^ total theca height. Basal plate diameter is 1/7^th^ total theca diameter.

Radial plates are higher than wide, and constitute more than ½ the height of the entire theca. Ambulacral lips are narrow, extending downward from theca, and tapering inward towards basals. Interradial seam is in a shallow depression, and is easily visible.

Radiodeltoids from the exterior of the theca in a slightly convex arc. Radiodeltoid suture is pointed, downward facing, and is flush with plates. Suture is located at about ½ height of theca. Theca periphery falls well below the suture, and is present about ¼ of the height of the theca. Deltoids at radiodeltoid suture exceed ambulacral width by about 3x. Deltoid is indented through the center, and tapers to an extended crest or comb orally. Where crests meet, theca comes to a point.

Slope to the ambulacral sinus is at a shallow angle, but falls to a somewhat deep groove. Ambulacral furrows are regular, about 14 in 5 mm, and fall in 2 inflated ridges running nearly the length of the theca. Individual furrows are linear to slightly curved. Overall, ambulacra are lancet shaped, and come to a rounded point basally and a sharp point orally.

Anal opening is large and teardrop shaped, easily discernible from the small, circular-to-ovoid oral openings.

### Remarks

*D*. *beaveri* sp. nov. has many features which set it apart from the many established species of *Deltoblastus*. *D*. *beaveri* has a radial length-to-deltoid length ratio in excess of 1, meaning it possesses elongate radials, atypical of all other *Deltoblastus* species [4]. The deltoid length-to-ambulacrum length ratio of *D*. *beaveri* sp. nov. is too small for it to conform to the standards set for *D*. *molengraaffi* [4] or *D*. *sebotensis* [4], which have a 3:1 or greater ratio. With wide ambulacra, *D*. *beaveri* sp. nov. does not conform to the thin lancet-shaped ambulacra or ornamentations present on *D*. *timorensis* [4], *D*. *globosis* [4], *D*. *somoholensis* [4], *D*. *jonkeri* [4], *D*. *delta* [4], *D*. *elongatus* [4], *D*. *subglobosus* [4], *D*. *magnificus* [4], *D*. *batheri* [4], or *D*. *pseudelta* [4]. The position of the periphery of *D*. *beaveri* is well below the radio-deltoid suture, indicating it is unlikely to be the same species as *D*. *verbeeki*, which commonly has the periphery at the radio-deltoid suture [4]. The elongate radials in this specimen set it apart from all other *Deltoblastus* species known.

### Occurrence

Permian of Timor.

***Deltoblastus ewini*** new species

urn:lsid:zoobank.org:act:E8C5BB71-E9F1-475C-8081-C5E0BA918208

(Fig 2D–2F)

### Derivation of name

Named for Dr. Timothy A. M. Ewin, Curator of Echinoderms, Natural History Museum of London.

### Type specimen

NHMUK e59727, e59734, e59209, e59210, and e59212.

### Diagnosis

*Deltoblastus ewini* sp. nov. is an obtusely globose species. *D*. *ewini* posseses shortened radials with a low-lying periphery, and pointed radiodeltoid suture falling above periphery. Pronounced inflation of deltoid ridges along ambulacral groove merges to become bladed peaks adorally. Long and wide petaloid ambulacra extend length of theca, and are strongly indrawn basally. Ornamentation absent.

### Description

Overall shape of *D*. *ewini* is extremely globose, tapering adorally. Theca not ornamented, excepting minor growth striae on a few specimens. Theca height is exceeded by theca width at greatest points, lending a globose shape to the theca. Anus is teardrop-to-oval shaped.

Radial length is greatly exceeded by deltoid length, ~1.6x. Deltoid 1.5 times wider than ambulacra at radio-deltoid suture.

Ambulacra are deeply indented and wide, with ten to twelve furrows in 5 mm of length. Ambulacra length is two times the deltoid length, and extend nearly the length of the theca. Ambulacra are petaloid in shape, wider towards the base of the theca, with basal ends indrawn. Oral end of ambulacra appear truncated, ending abruptly at oral surface.

Radials and deltoids produce raised ridges along length of ambulacra, and are slightly depressed through the center of the plates. Orally, deltoids become pointed peaks or crests. Radio-deltoid suture is pointed in a v-shape towards the basals, and is placed at or slightly above the periphery. Deltoids are triangular in shape, widest at the radio-deltoid suture. Elevation of deltoids above ambulacra is greatest near oral surface.

Overall, radial plates are strongly convex. Ambulacral extension into radial plate nearly bifurcating, with 2/3 dominated by ambulacral presence. Radial-radial suture barely visible, creating slight depression.

Basal plates are small, and only shallowly invaginated.

### Remarks

The extreme globosity of this species sets it apart from other named species of *Deltoblastus*. Initial observation of *D*. *ewini* sp. nov. would lead to the diagnosis of *D*. *globosus* [4]; yet, the pointed radio-deltoid suture, which is more akin to that observed on *D*. *jonkeri* [4], excludes this diagnosis. *D*. *jonkeri*, with an average radial/deltoid length ratio of greater than 0.5 [4], possesses radials that are too long and is, on average, too elongate in overall shape to be a viable alternative species assignation.

### Occurrence

Neoetpantoekak and Basleo localities, Sonnebait Series, Timor, Permian.

***Deltoblastus elevatus*** new species

urn:lsid:zoobank.org:act:1C241EE2-76ED-4CB3-AD57-C38AC7E27122

(Fig 2G–2I)

### Derivation of name

Named for the extreme length of the deltoids; from Latin *elevo*, meaning elevated.

### Type specimen

USNM 594946.

### Paratype

USNM 594947.

### Diagnosis

*Deltoblastus elevatus* sp. nov. possesses an extremely elongate, ellipsoidal theca. Ambulacral indentation is shallow, with wide, petaloid ambulacra extending the length of the theca. Deltoids are extremely elongated for *Deltoblastus* and narrow. Radio-deltoid suture horizontal, and is placed well below the periphery. Radials short, less than half the length of deltoids. Deltoid length greater than 4.5x deltoid width at radio-deltoid suture.

### Description

Overall form of *Deltoblastus elevatus* resembles an elongate ellipsoid, which very slightly tapers adorally. Theca width is half that of theca height at greatest points. Theca lacks ornamentation and growth striae. Deltoid length exceeds radial length by a factor of 2.

Deltoid width is equal to ambulacral width at radio-deltoid suture. Deltoids elongate, and slightly tapering orally. Deltoid length exceeds deltoid width at greatest points by a factor of 4.5. Deltoids are raised above ambulacral surface, creating a narrow, steep-sided platform. Deltoids sharply indrawn at oral surface and produce narrow crests.

Ambulacra are shallowly indented and wide, with ambulacral side furrows in excess of 13 per 5 mm of length. Side furrow surface is slightly convex. Ambulacral length is 1.3x that of deltoids, with ambulacra extending the length of the theca. Radials and deltoids produce a raised platform along the length of the ambulalcra.

Radio-deltoid suture near horizontal, with periphery well above. Radials short, strongly influenced by ambulacral extension. Radial plates sharply convex, almost at right angle. Radial-radial suture is barely visible, flush with plate surface.

Reduced, invaginated basal plates are slightly depressed from radial surface. Radial-basal plate margin sharply contrasts with radial platform.

### Remarks

*D*. *elevatus* sp. nov. is similar in basic form to *D*. *ellipticus*, with ambulacral side furrows in excess of 13 per 5 mm of length; however *D*. *elevatus* possesses extremely elongate deltoids, whose length is greater than 4.5x the width of the deltoids at the radio-deltoid suture, far in excess of the 2.4x-2.8x required for *D*. *ellipticus*, or the 3x required for *D*. *elongatus* [4]. No other *Deltoblastus* species displays such distinctly elongate deltoids or morphology.

### Occurrence

Basleo locality, Timor, Permian.

## Conclusions

*Deltoblastus* morphology is complex but defining characters important to species differentiation can be resolved using simple visualization techniques. This paper used data taken from the original works on *Deltoblastus* species to establish a character matrix that can be used to ascertain species affinities based on simple metrics. Three new species of *Deltoblastus* are resolved using this approach, with each demonstrating a unique morphology. *Deltoblastus beaveri* is unique in its possession of shortened deltoids and elongated radials. *Deltoblastus ewini* demonstrates extreme globosity, with shortened radials and wide ambulacral grooves. Finally, *Deltoblastus elevatus* displays the opposing extreme, with pronounced elongation of the theca, especially of the deltoid plates.

## References

[1] Waters JA. The paleobiogeography of the Blastoidea (Echinodermata). In: McKerrow and Scotese, editors. Palaeozoic Palaeogeography and Biogeography. The Geological Society Memoir. 1990; 12, pp. 339–352.

[2] Charlton TR, Barber AJ, Harris RA, Barkham ST, Bird PR, Archbold NW, et al The Permian of Timor: Stratigraphy, paleontology and palaeogeography. Journal of Asian Earth Sciences. 2002; 20: 719–774.

[3] Teichert C. The Marine Permian Faunas of Western Australia. Palaontologische Zeitschrift. 1951; 24, pp. 76–90.

[4] Webster GD, Sevastopulo GD. Paleogeographic significance of Early Permian crinoids and blastoids from Oman. Paläontologische Zeitschrift. 2007; 81, pp. 399–405.

[5] Fay RO. *Deltoblastus*, a new Permian blastoid genus from Timor. Oklahoma Geology Notes. 1961; 21(2), pp. 36–40.

[6] Jansen H. Die Variationsstatistische methode angewandt auf ein groszes material van *Schizoblastus* aus dem Perm van Timor und einige neue a nomalien dieser Gattung. Koninklijke Nederlandse Akademie Van Wetenschappen. 1934; 37(10), pp. 819–825. German.

[7] Wanner J. Die Permischen Echinodermen von Timor: Palaontologie von Timor. 1924. Teil II, Leiferung XIV, Abh. XXIII, pp. 1–81, pls. 199–206, Fig 1–31, Stuttgart. German.

[8] Wanner J. Die Permischen Blastoiden von Timor. Jaarbook van het Mijnwezen in Nederlandsch Oost Indie, 1922. 1924; 51, pp. 163–233. German.

[9] Bather FA. Jungeres Palaozoikum von Timor, Genus Schizoblastus Etheridge and Carpenter. In Boehm G, editor. Geologische Mittleilungen aus dem Indo-Australischen Archipel. Neus Jahrb. Mineralogie, usw., Beil.-Band, 25. 1908. pp. 303–319, pl. 10–11. German.

[10] Say T. On Two Genera and Several Species of Crinoidea. Journal of the Academy of Natural Sciences of Philadelphia. 1825; 1(4), pp. 289–296.

[11] Etheridge RJr, Carpenter PH. Catalogue of the Blastoidea in the Geological Department of the British Museum (Natural History) with an account of the morphology and systematic position of the group, and a revision of the genera and species. British Museum Catalogue. 1886. 332 p.

